# Atypical presentation of cystic schwannoma of the sphenoid sinus: a nonsolitary mass with osseous, intracranial and cavernous sinus invasion

**DOI:** 10.11604/pamj.2018.31.233.16515

**Published:** 2018-12-19

**Authors:** Gautam Dutta, Daljit Singh, Hukum Singh, Ghanshyam Singhal, Ravindra Kumar Saran

**Affiliations:** 1 Department of Neuro-Surgery, Govind Ballabh Pant Institute of Postgraduate Medical Education and Research (GIPMER), New Delhi, India

**Keywords:** Cystic schwannoma, sphenoid sinus, paranasal sinus, mucocele

## Abstract

Although nearly half of all schwannomas involve the head and neck region, nasal and paranasal sinus presentations are quite rarely seen. Cystic schwannoma, characterized by cyst formation lined by S-100 protein positive membrane-like structures is very uncommonly seen in sphenoid sinus with only a single previously reported case. Here we report a young patient of cystic schwannoma of the paranasal sinuses having epicenter in the sphenoid sinus. The tumor had caused extensive erosion of the skull base and paranasal sinuses and extended intracranially that radiologically mimicked as infected mucocele causing loss of vision. This case denotes the aggressive behavior of such uncommon tumors.

## Introduction

Schwannomas are slow growing benign tumors originating from the peripheral nerve sheaths. Approximately 25-50% of schwannomas occur in the head and neck region, but tumors originating from the nasal cavity or paranasal sinuses are rare, with just over 100 cases reported in literature [1]. Cystic schwannoma (CS), recognized by cyst formation lined by S-100 protein positive membrane-like structures in an otherwise ordinary schwannoma [2], is very unusually seen in paranasal sinuses. Till now, only a single case of CS of the sphenoid sinus has been described in literature [3]. Here we present an unusual case of CS of the paranasal sinuses having epicenter in the sphenoid sinus that masqueraded as an infected mucocele. The tumor had caused extensive erosion of skull base and paranasal sinuses and grown intracranially causing unilateral loss of vision in a young adult. This case is one noteworthy example of the aggressive behavior of such uncommon tumors.

## Patient and observation

**Figure 1:**
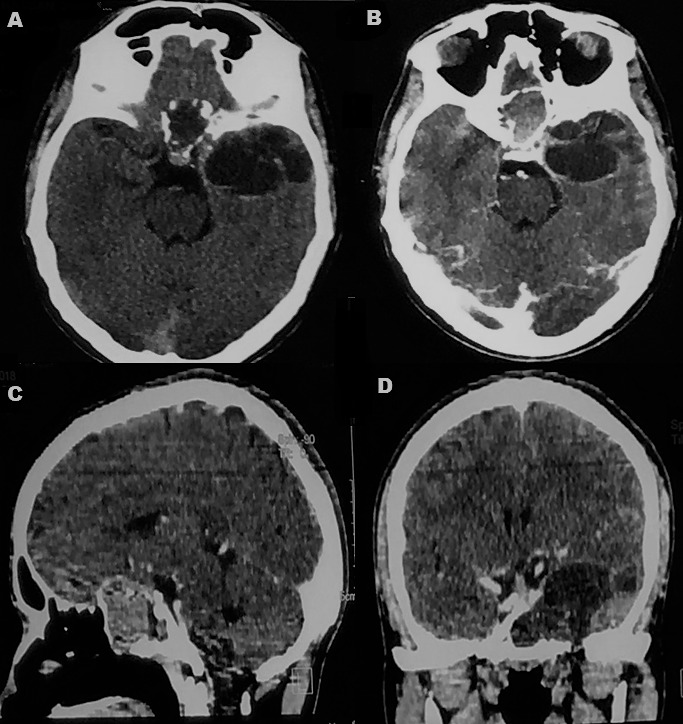
(A-D) CECT brain and paranasal sinuses showing infiltrative solid-cystic mixed density lesion epicentred in the base of skull predominantly involving the sphenoid sinus causing its expansion and ballooning. The lesion has caused erosion of the left lateral wall of the sphenoid sinus, left anterior and posterior clinoid process, floor of sella and sphenoid sinus and lateral wall of the left orbit with extension into the left antero-medial temporal lobe and left cavernous sinus

**Figure 2:**
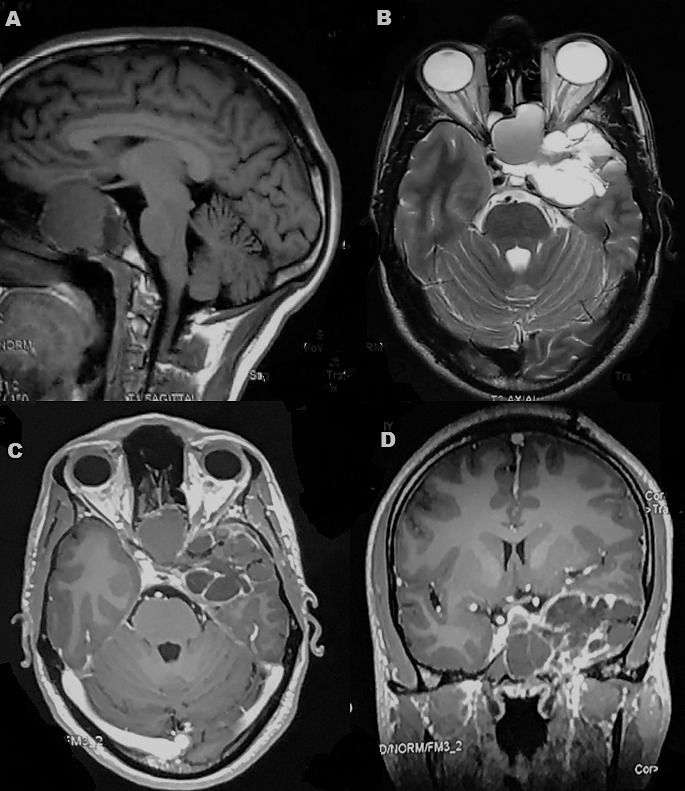
(A-D) CEMRI brain and paranasal sinuses showing a multiseptated cystic lesion appearing iso to hyperintense on T1 and T2W images causing cortical buckling and displacement of the left temporal lobe. Peripheral enhancement of the lesion is evident on contrast

**Figure 3:**
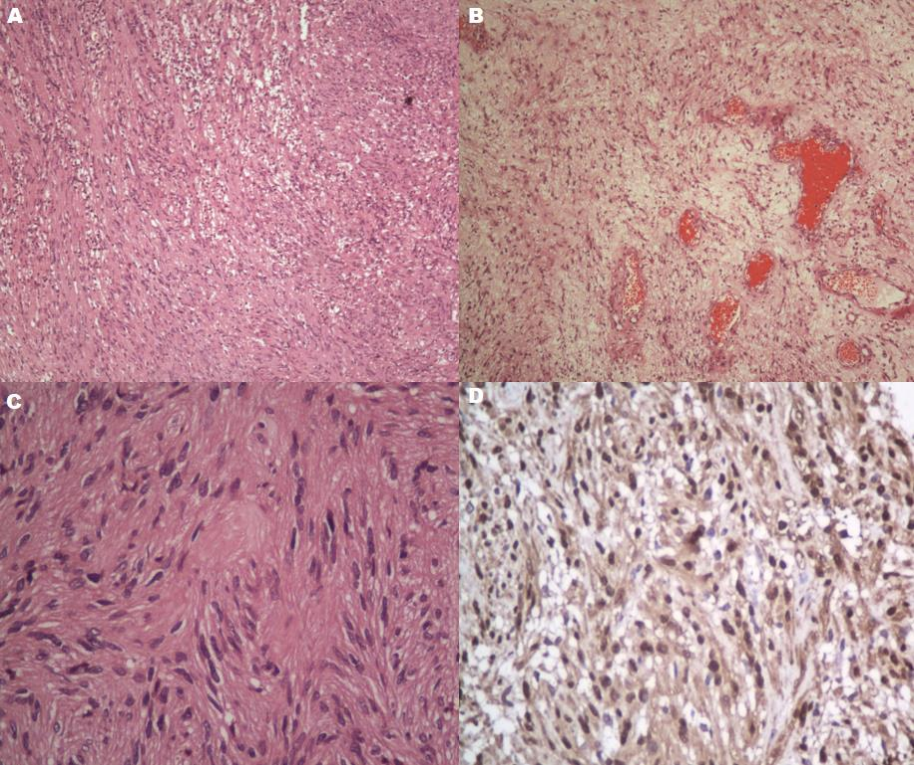
H&E x 40 showing a spindle cell neoplasm, with hypercellular areas representing Antoni (A) and edematous hypocellular areas of Antoni (B) type and palisading nuclei consistent with Verocay bodies (C). On immunohistochemistry (D), tumor cells and membrane lining the cyst is positive for S-100 protein (magnification x 40)

A 22-year old male was seen in our neurosurgical unit having complaints of dull-aching type of holocranial headache since past 6 months and gradually progressive painless diminution of vision of the left eye since past 2 months. He did not give any history of diplopia, nasal obstruction or epistaxis. Neuro-ophthalmologic examination revealed visual acuity of 6/6 in right eye and only light perception in the left eye along with relative afferent pupillary defect and features of optic atrophy on the left side. Neurological examination was otherwise unremarkable. Contrast-enhanced computed tomography (CECT) brain and paranasal sinuses were obtained which revealed a large infiltrative solid-cystic mixed density lesion epicentred in the base of skull predominantly involving the sphenoid sinus causing its expansion and ballooning. The lesion had caused erosion of the left lateral wall of the sphenoid sinus, left anterior and posterior clinoid process, floor of sella and sphenoid sinus and lateral wall of the left orbit with extension into the left antero-medial temporal lobe and left cavernous sinus (Figure 1). On contrast-enhanced magnetic resonance imaging (CEMRI), the lesion was multiseptated and cystic, appearing iso to hyperintense on all the sequences and having no restriction on diffusion. The lesion showed cortical buckling and displacement of the left temporal lobe. The lesion was also seen extending into the left masticator space through the left pterygopalatine fissure which appeared widened with resultant erosion of the lateral wall of the left maxillary sinus. The left cavernous sinus was having >180 degrees encasement of the left cavernous internal carotid artery. Peripheral enhancement of the lesion was evident on contrast administration. The findings were suggestive of infected mucocele. (Figure 2). The endoscopic biopsy of the sphenoid sinus mass was attempted first by a head and neck surgeon which however returned a bloody aspirate and the cytological examination was inconclusive. The culture did not reveal any growth either. A left pterional craniotomy with anterior ethmoidectomy (Lynch Howarth approach) was performed and tumor was decompressed. Intraoperatively, tumor was found to be present extra- and inter-durally in the left temporal fossa, filling the sphenoid and ethmoid sinuses. Tumor was soft and highly vascular, capsulated and having well-defined cleavage plane. A near-total excision was achieved. Histopathological examination of the resected specimen revealed a spindle cell neoplasm, with hypercellular areas corresponding to Antoni A (Figure 3, A) and edematous hypocellular areas of Antoni B type (Figure 3, B). Palisading nuclei consistent with Verocay bodies were noted (Figure 3, C). No atypical nuclear features, atypical mitosis or necrosis were seen. Mitotic rate was less than 1/10 high power field (HPF). There were hyalinized blood vessels and some of them were obliterated along with intratumoral hemorrhage and pigmented macrophages. Tumor was surrounded by a thick fibrocollagenous capsule and the cystic space was lined by a thin eosinophilic membrane. Tumor cells were positive for S-100 protein and vimentin and negative for epithelial membrane antigen (EMA) and cytokeratin (Figure 3, D). S-100 protein was also found to be strongly positive at the membrane lining the cyst. The histopathology was consistent with CS. Postoperatively the patient improved well although the visual acuity remained same as preoperative state. He is currently on regular follow up since past 6 months.

## Discussion

Schwannoma is a painless, benign slow growing tumor of the peripheral nerve sheath commonly associated with the cochlear nerve at the cerebellopontine angle. They rarely occur in the sinonasal cavities and in this location, they are typically described as solitary encapsulated lesions confined to the nasoethmoid region [4]. Schwannomas originate from Schwann cells of the peripheral nervous system. There have been various hypotheses proposed to explain the origin of intracranial schwannomas that do not arise from cranial nerves. Schwann cell hyperplasia is the most commonly proposed theory, occurring within the perivascular nerve plexuses [5], the meningeal branch of the trigeminal nerve in the anterior cranial fossa, the anterior ethmoidal nerve around the cribriform plate, or the fila olfactoria, which are known to acquire Schwann cell sheaths that extend ~0.5 mm beyond the olfactory bulb [6]. The origin of schwannomas arising from the paranasal sinuses are presumed to be from the peripheral nerve sheaths of the branches of the ophthalmic and maxillary divisions of the trigeminal nerves and their ganglion and lack of any other nerve divisions near these sinuses. The sphenoid sinus is richly innervated by the posterior ethmoidal nerve, and we surmise that the current case might had its origin from this nerve. Cystic change or degeneration may be encountered in schwannomas frequently which is somehow a feature of so called “ancient schwannoma”, which is also well known for atypical nuclear features of no particular prognostic significance. But in the case of ancient schwannoma, cystic areas are smaller, multiple and are not lined by S-100 protein positive membrane-like structures [7]. CS are recognized by a typical clinical history of subtle complaints lasting for a long time, followed by acute progressive symptoms. This course is reminiscent of malignant transformation in a schwannoma [8]. In our patient, however, the complaints were not of very long duration (6 months), however the acute progressive nature of symptoms were suggested by his vision loss which occurred within a short period of time. It has been suggested that the accelerated clinical findings are not related to sudden tumor growth due to cell proliferation, but are due to expansion of the cyst [2]. It is believed that the clinical outcome of CS is worse than solid tumors [2]. However, it is difficult to assess this for cases occurring at the paranasal sinuses, since there are few cases of CS reported. For the present case total excision was the treatment of choice and the patient is in good health 6 months after the operation without any evidence of recurrence. Based on the current case, we can presume that maximum safe resection should be the primary goal of surgical intervention. However, long duration follow up is needed to better assess the clinical behavior of such uncommon tumors of paranasal sinus.

## Conclusion

Our patient's schwannoma, presumed to originate from the posterior ethmoidal nerve at the sphenoid sinus, appears to be unique in its presentation as an erosive fronto-ethmoidal mucocele and loss of vision and a location which is very uncommonly seen. The current case is one noteworthy example of mucocele formation secondary to the underlying schwannoma which may mimic features of the more commonly encountered lesions in this region that may cause diagnostic dilemmas and give intraoperative surprises. It remains to be seen if such unusual tumors can be studied in a more extensive way in their earlier juncture of development when the tumors are still small and if with the new radiological techniques like high-tesla MRI the nerves of their origin can be revealed and these tumors subclassified.

## Competing interests

The authors declare no competing interest.

## References

[1] Gencarelli J, Rourke R, Ross T (2014). A typical presentation of sinonasal cellular schwannoma: a nonsolitary mass with osseous, orbital and intracranial invasion. J Neurol Surg Rep.

[2] Charabi S, Tos M, Thomsen J, Rygaard J, Fundova P, Charabi B (2000). Cystic vestibular schwannoma-clinical and experimental studies. Acta Otolaryngol.

[3] Di Nardo LJ, Mellis MG (1993). Cystic schwannoma of the sphenoid sinus and skull base. Ear Nose Throat J.

[4] Suh JD, Ramakrishnan VR, Zhang PJ (2011). Diagnosis and endoscopic management of sinonasal schwannomas. ORL J Otorhinolaryngol Relat Spec.

[5] Nelson E, Rennels M (1970). Innervation of intracranial arteries. Brain.

[6] Li YP, Jiang S, Zhou PZ, Ni YB (2011). Solitary olfactory schwannoma without olfactory dysfunction: a new case report and literature review. Neurol Sci.

[7] Nakayama H, Gobara R, Shimamoto F, Kajihara H (1996). Ancient schwannoma of the oral floor and ventricular portion of the tongue: a case report and review of the literature. Jpn J Clin Oncol.

[8] Woodruff JM, Selig AM, Crowley K, Allen PW (1994). Schwannoma (neurilemoma) with malignant transformation: a rare, distinctive peripheral nerve tumor. Am J Surg Pathol.

